# Effects of Non-Pharmacological Treatment on Pain, Flexibility, Balance and Quality of Life in Women with Fibromyalgia: A Randomised Clinical Trial

**DOI:** 10.3390/jcm10173826

**Published:** 2021-08-26

**Authors:** Juan Rodríguez-Mansilla, Abel Mejías-Gil, Elisa María Garrido-Ardila, María Jiménez-Palomares, Jesús Montanero-Fernández, María Victoria González-López-Arza

**Affiliations:** 1 ADOLOR Research Group, Department of Medical-Surgical Therapy, Faculty of Medicine and Health Sciences, Extremadura University, 06006 Badajoz, Spain; jrodman@unex.es (J.R.-M.); abel_mejias@hotmail.com (A.M.-G.); mariajp@unex.es (M.J.-P.); mvglez@unex.es (M.V.G.-L.-A.); 2 Mathematics Department, Faculty of Medicine and Health Sciences, Extremadura University, 06006 Badajoz, Spain; jmf@unex.es

**Keywords:** fibromyalgia, exercise for well-being, active exercise program, flexibility, static balance, pain, quality of life

## Abstract

Background: The functional deficits in people with fibromyalgia can be related to the level of physical activity performed. This study investigated the effectiveness of an active exercise programme versus exercise for well-being improving pain, flexibility, static balance, perceived exertion and quality of life of women with fibromyalgia; Methods: A randomised, single-blind, controlled trial was conducted. A total of 141 of women diagnosed with fibromyalgia were enrolled and randomised to an active exercise program group (*n* = 47), where they performed physical active exercises, an exercise for well-being group (*n* = 47), which performed the Qi Gong exercises named ‘the twenty Wang Ziping figures for health and longevity’, and a control group (*n* = 47), which did not receive any intervention, for a period of 4 weeks. Measures were taken at baseline and after the treatment. The primary outcome measures were static balance and centre of gravity (Wii-Fit Nintendo ©), flexibility (test de Wells and Dillon), pain (Visual Analogue Scale) and quality of life (Spanish-Fibromyalgia Impact Questionnaire). The secondary outcome measure was the perceived exertion during activity (BORG Scale). Results: In total, 93 participants completed the study. The mean value of the age was 52.24 ± 6.19. The post intervention results showed statistically significant improvements in the exercise for well-being and the active exercise programme groups vs. the control group in relation to pain (*p* = 0.006 active exercise programme group, *p* = 0.001 exercise for well-being group), static balance (*p* < 0.001 active exercise programme group) and quality of life (*p* < 0.001 active exercise programme group, *p* = 0.002 exercise for well-being group). In addition, the mean scores related to perceived fatigue during the sessions were 6.30 ± 1.88 for the active exercise programme group and 5.52 ± 1.55 for the exercise for well-being group. These differences were not significant. Conclusions: The active exercise program and exercise for well-being improved flexibility, static balance, pain and quality of life of women with fibromyalgia. The participants of the active exercise programme achieved better results that those of the exercise for well-being.

## 1. Introduction

The main clinical manifestation of Fibromyalgia is diffuse and widespread pain in combination with the presence of multiple tender points [1]. In addition to pain, these patients have sensory symptoms, such as paraesthesia, motor symptoms, such as muscle stiffness, contractures and tremors, and vegetative symptoms, such as tingling sensations [2].

Different authors have suggested that these symptoms can affect the functional capacity of these patients [3,4]. This is based on the association between symptoms, flexibility and balance impairments [3,4]. Moreover, balance impairment is a very frequent sign in persons with fibromyalgia and it is considered one of the 10 most disabling symptoms, with a prevalence between 45% and 68% [5]. In addition, it has been shown that these impairments often appear in persons with the same conditions, such as chronic fatigue syndrome, especially the loss of static and dynamic balance [6], which can lead to impaired mobility [7]. Vestibular function may be impaired in patients with chronic fatigue syndrome who also have fibromyalgia but not in those with chronic fatigue syndrome alone [8].

A study conducted by Jones et al. [5] showed how persons with fibromyalgia had significant inferior scores on different balance aspects and had six times more falls when compared with healthy subjects. Balance impairments and functional capacity are closely related [9] and have a significant impact in the quality of life of people with fibromyalgia [2].

These abilities are diminished or altered in patients with fibromyalgia compared to healthy subjects [10,11,12,13,14]. This can lead to limited or difficult mobility, which can increase the risk of falls [15,16], and consequently, it can have a negative impact on the quality of life of these patients [2].

According to the scientific evidence, these functional deficits in people with fibromyalgia are related to the level of physical activity performed [9]. Several systematic reviews analyse the efficacy of physical exercise programmes, either alone or in combination with other forms of physical or cognitive intervention [17,18,19]. All of them conclude that physical exercise improves the quality of life of these patients. In this regard, a literature review on the benefits of exercise in fibromyalgia published in 2019 [20] concluded that exercise also improves physical function and fatigue. However, further studies and research are needed to analyse this further [20].

Complementary and alternative therapies are currently being used as a non-pharmacological intervention for the management of fibromyalgia [21]. The World Health Organisation defines exercise for well-being (Qi Gong) as: “A component of traditional Chinese medicine that combines movement, meditation and breathing regulation to improve the flow of vital energy in the body (Qi), to improve circulation and immune function” [22].

The available literature supports that exercise for well-being improves pain management [23,24] and physical function [24] in patients with fibromyalgia. In addition, some clinical trials have shown that this treatment technique also improves balance and prevents falls [25]. Qi Gong is an aerobic exercise, which involves mental concentration, breathing that accompanies the movement, static postures and dynamic movements which combine stretching and activation of the muscle chains through isometric and isotonic contractions. It also includes self-massage movements and flexibility, strength, proprioception, coordination and balance work [26,27,28]. Qi Gong also corrects the posture of the spine and the pelvis and prevents stagnation of the energy in the joints [29]. On this basis, scientific research suggests that low-intensity aerobic exercise and meditative movement therapies, such as Qi Gong, are recommended for the treatment of fibromyalgia patients, as they improve their symptoms and quality of life [30,31,32].

However, the research conducted on this topic is scarce and the existing studies agree that further research on the effects of these alternative therapies in patients with fibromyalgia is needed. In the literature consulted, no studies that analyse these variables and compare both treatments, physical exercise or an active exercise programme and exercise for well-being, have been found.

Based on all this, the aim of this study was to evaluate the effectiveness of an active exercise programme and exercise for well-being exercise programme improving pain, flexibility, static balance and quality of life in patients with fibromyalgia, comparing both treatment approaches between them and with a control group.

## 2. Materials and Methods

This was a single-blind randomised clinical controlled trial. The CONSORT statements were used to conduct and report the trial. Ethical approval was granted by the Bioethical Commission of the University of Extremadura in Spain (Reference number: 11/2012). The trial was retrospectively registered with the ClinicalTrials.gov registry (Study Identifier: NCT04328142). All the participants signed a written informed consent prior to their participation in the study.

The target population was women diagnosed with fibromyalgia from the Fibromyalgia Associations from Badajoz and Olivenza in Extremadura (Spain). The recruitment period took place from March to October 2012.

The inclusion criteria were: women between 30 and 65 years old, diagnosed with fibromyalgia [1] by a specialised physician at least one year before the study began. Potential participants were excluded if they had been prescribed with active exercise treatment previous to the study, they did regular physical exercise or aerobic training, they had previous knowledge of exercise for well-being or they had mobility impairments or absence of any limb.

**Figure 1:**
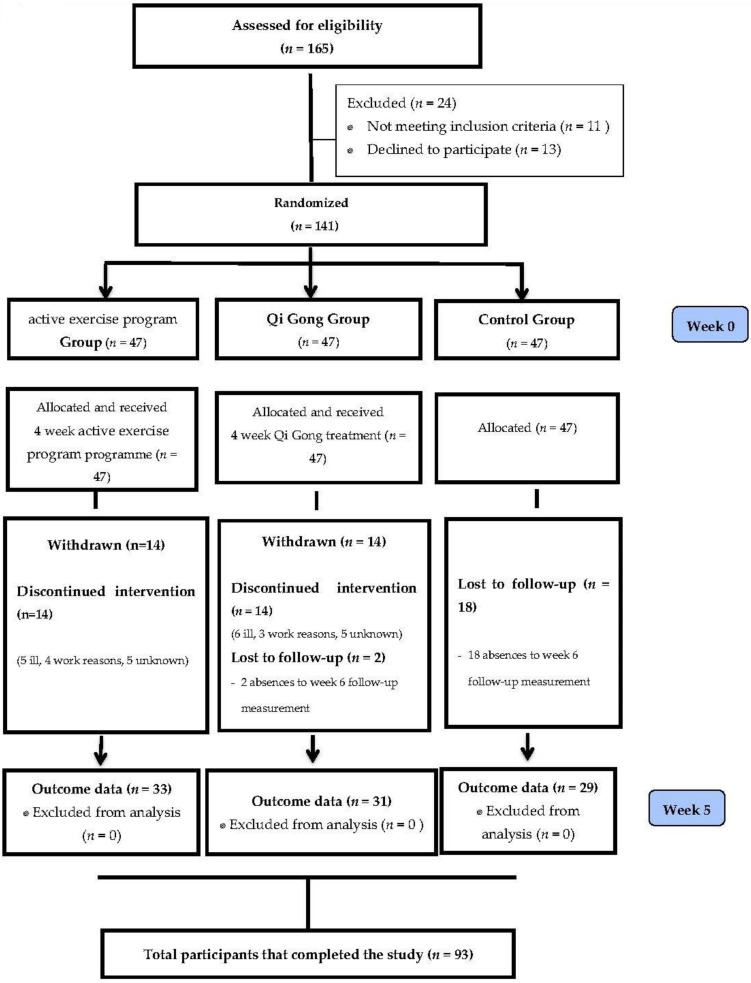
Flow diagram of study participation.

An independent researcher who was unrelated to any aspect of the trial was responsible for the randomisation. A total of 141 participants were randomly allocated to an active exercise programme experimental group, an exercise for well-being experimental group or a control group (Figure 1). A total of 141 sealed envelopes containing the group names were put in an opaque bag. The independent researcher kept the bag closed during the randomisation process. The participant was in charge of opening the bag and the envelope during this process. After the first assessment, the researcher informed the participants to which group they were allocated to. The allocation of each participant was concealed at all times until assignment. No one directly involved in the study had access to the randomisation process or the list.

The study was conducted over six weeks: four weeks of treatment and two weeks of measurements. All participants were requested to attend two measurement sessions: the baseline assessment and the post intervention assessment. The University of Extremadura laboratories were the location where all measurement sessions took place. The assessor was blinded to the group allocation. He was independent to the study and was not aware of the treatments applied. Neither the participants nor their therapists were blind to the group assignment. Due to the nature of the treatment, they could clearly see to which group the participant was allocated.

The following variables were measured through a data collection protocol: sociodemographic data: age, education, working status and marital status.

Outcome measures: The primary outcome measures were static balance, flexibility, pain and quality of life. The secondary outcome measure was the perceived exertion during activity. The measurement tools used were as described below.

Balance test: A plantar pressure platform with optical sensors (Wii-Fit Nintendo ©) was used to assess balance. The patients, standing on the platform and with their feet on the specified marks, had to maintain a standing posture while their centre of gravity was being recorded. The displacements to the left and right were assessed as deviations in percentages. Subsequently, stabilometry was carried out by means of the one-leg stand test with a duration of 30 s. The value in percentage (0–100%) of their stability was obtaining with this test. The higher the value achieved, the better the balance.

Wells and Dillon *Test* *or Sit and Reach Test:* This test assesses the trunk flexion flexibility [33]. It has a relative intra-examiner reliability (0.89–0.99) and moderate validity that oscillates between r = 0.37–0.77 for men and r = 0.37–0.85 for women [34]. This test is performed with the aid of a measuring box which has on its front the numerical measurement values that correspond to a metre.

The patient is placed in a sitting position on the measuring box with feet together at a right angle. In this position, the patient is asked to make a maximum flexion of the trunk, with the knees extended and the upper limbs in full extension, using the palms of the hands and pushing a ruler until they have reached the maximum possible distance. The distance achieved by pushing with the fingers is measured in centimetres. As the patient moves away from zero, the centimetres achieved are noted with a positive sign. If, on the other hand, the person does not reach the tip of the toes, the remaining centimetres to zero are marked with a negative sign. The higher the positive value, the better the results. We quantified the improvement as the greater number of centimetres achieved.

Visual Analogue Scale (VAS) of pain: This scale is a valid and reliable measure for the assessment of pain. It has proved its validity with high correlations with other pain measures (r = 0.62 to 0.91) and its reliability with a good test-retest (r = 0.94 to 0.71) [35]. Participants were asked to rate their worst pain intensity during the last week using a 100-mm VAS, with 0 denoting “no pain” and 100 denoting “extreme and unbearable pain” [36].

Quality of life: The impact of the condition on the patient’s quality of life was assessed with the Spanish Fibromyalgia Impact Questionnaire (S-FIQ) [37]. This is the Spanish adaptation of the Fibromyalgia Impact Questionnaire [38]. The S-FIQ has a reliability coefficient of 0.81. The maximum score is 100 and the higher the result obtained, the higher the impact of the condition on the person.

Borg Scale of Perceived Exertion: This scale is a very useful tool to measure the perceived effort made in an activity. The Borg Scale of Perceived Exertion has an acceptable validity and reliability. Correlation coefficients between scale scores and heart rate, as well as test and post-test, are greater than 0.70 [39]. It consists of 10 numerical levels of dyspnoea ranging from 0 to 10 points: 0, rest; 1, very mild; 2, mild; 3, moderate; 4, somewhat hard; 5 and 6, hard; 7, 8 and 9, very hard; 10, maximum [40].

The sample was allocated to three groups: the experimental active exercise programme group, which completed an active physical exercise treatment programme, the experimental exercise for well-being group, which received exercise for well-being treatment, and the control group, which did not receive any treatment. Each group had 47 participants.

The study was conducted over 6 weeks: 4 weeks of treatment and 2 weeks of assessments. The measurements were done at baseline, the week before the beginning of the treatments and post intervention, the week after the treatments were completed.

The participants that were allocated to the active exercise programme group completed an active exercise programme, which was guided by a qualified physiotherapist, who is a member of the Spanish Chartered Society of Physiotherapists and is trained in exercise for fibromyalgia. The exercise programme aimed to work on all the musculoskeletal system. Therefore, it included a warm up of 3 to 5 min of walking, active mobilisation exercises of the shoulders, spine and hips, static balance exercises and stretches. The shoulder, hip and cervical spine exercises were performed in a standing posture. The thoracic spine and lumbar spine were done on an exercise mat. All exercises were performed in coordination with controlled gentle breathing. Each mobilisation exercise was done at maximum range of movement, was maintained for 10 s and repeated six times with eyes open and closed. All movements were done slowly and pain and fatigue were avoided.

The exercise for well-being was guided by an exercise for well-being teacher with 20 years of experience and qualified by the International Institute of Exercise for Well-Being (funded by Yes Requena). The exercises performed during the sessions were the ‘twenty Wang Ziping figures for health and longevity’. These exercises are based on centennial therapeutic exercises from Daoyin, Wiqinxi, Yijinjing and Baduanjin, which are transmitted orally from master to disciple. The figures combine mental concentration and abdominal breathing during the performance of balance, flexibility and coordinated body movements. Each figure was repeated six times.

The active exercise program sessions as well as the exercise for well-being sessions lasted for 45 min and were done twice a week. The control group did not receive any intervention. All participants continued with their routine medical complying with the beneficence and non-maleficence principles of bioethics. More detailed information on the exercise programmes can be found in Figures S1–S3.

### Statistical Analysis

The sample size did not respond to a previous calculation since as many subjects as possible were recruited. Finally, approximately 30 participants could be randomly assigned to each experimental group. As a reference, with this sample size and for a significance level of 5%, a minimum power of 80% could be achieved if we aimed to detect an effect size of 0.5 by a *t*-paired test.

The sociodemographic characteristics of the patients were analysed and described. The baseline values of the main outcome measures were also described by groups. A one-way ANOVA was applied to verify the homogeneity of the three experimental groups. For each main outcome, a comparison of the evolution by group was carried out by a repeated measures model, considering the group (control group, active exercise group and exercise for well-being group) as an inter-group factor and pre-post outcomes as an intra-group factor. We focused on interaction results so that, when it was not significant, the comparison between groups was analysed. When it was significant, the Tukey HSD post hoc comparison for the full model was applied and significant results were highlighted (taking into account that, since it involves 15 different contrasts, it is a conservative procedure that tends to provide no significant results with samples of moderate size). Additionally, the size effect for interaction (partial η^2^) was reported.

The correlations between the age and each of the main outcome measures were analysed and the correlation test was applied. Student’s independent samples test was applied to conduct other contrasts with just two means involved. The analysis was performed with SPSS version 22 and jamovi 1.8.4.

## 3. Results

A total of 93 participants completed the study. The active exercise programme group had 33 participants, the exercise for well-being group had 31 and the control group had 29. During the intervention and the follow-up period, there were a total of 48 withdrawals. The corresponding data were excluded from the statistical analysis. A CONSORT flow diagram is given in Figure 1.

The mean value of age was 52.24 ± 6.19. The youngest woman in the study was 34 and the eldest was 65. As expected, age showed a significant correlation with the baseline scores in flexibility, as this outcome measure worsened with age. Nevertheless, we hardly found significant correlations between age and changes along the treatment (except for flexibility, which got better with age). The rest of the sociodemographic variables are described in Table 1.

**Figure 2:**
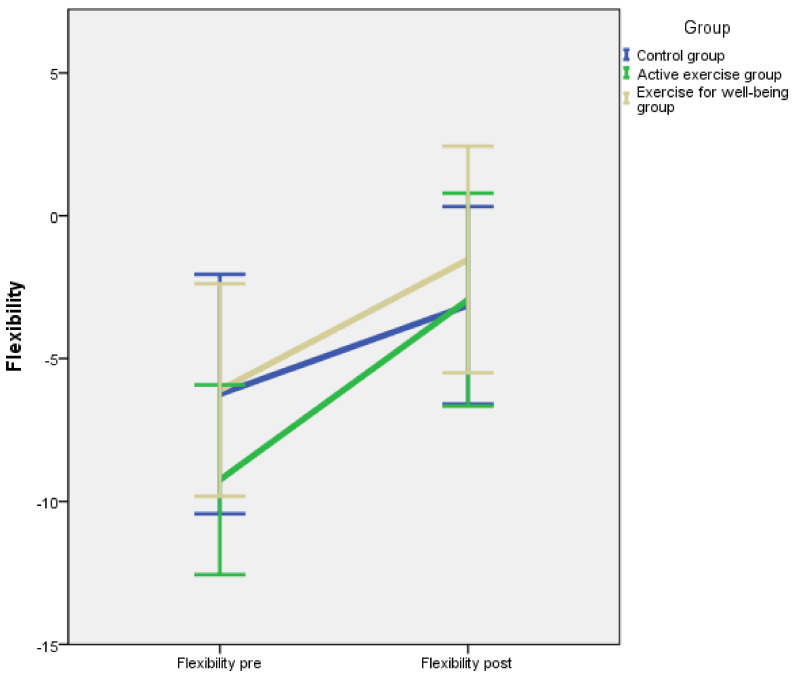
Changes in Flexibility.

**Figure 3:**
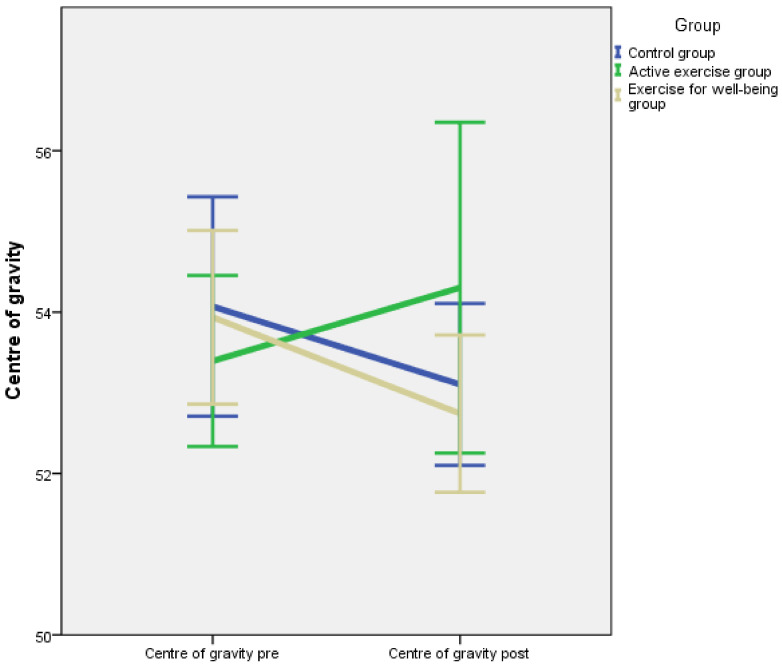
Changes in the centre of gravity.

**Figure 4:**
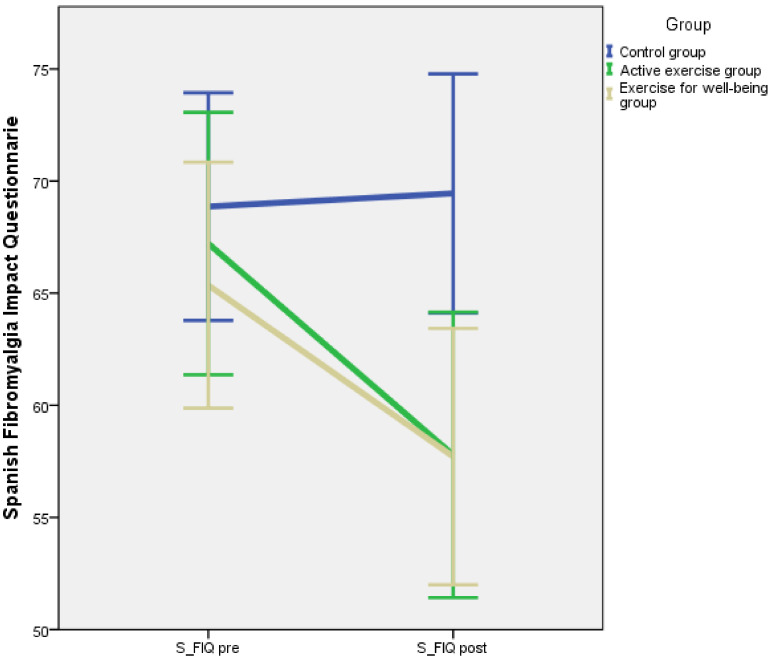
Changes in the Spanish Fibromyalgia Impact Questionnaire.

**Figure 5:**
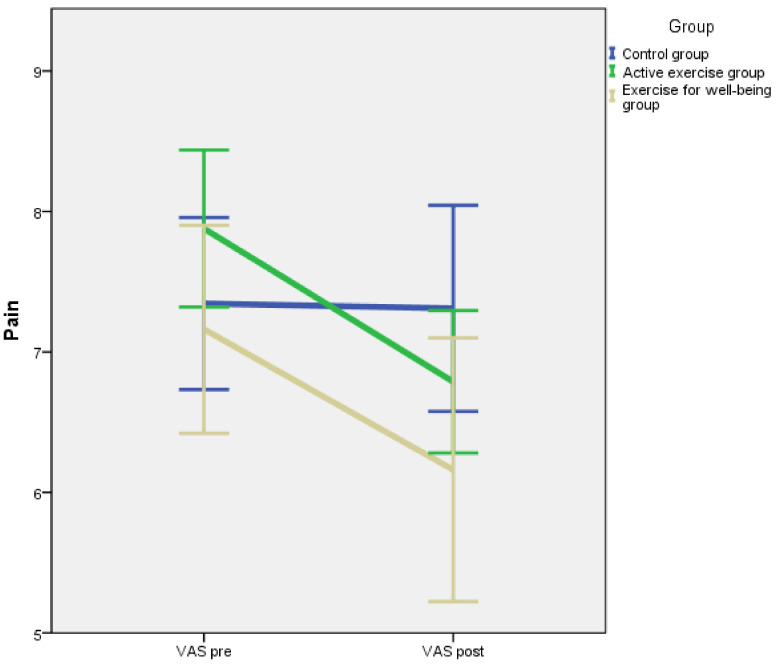
Changes in pain.

**Figure 6:**
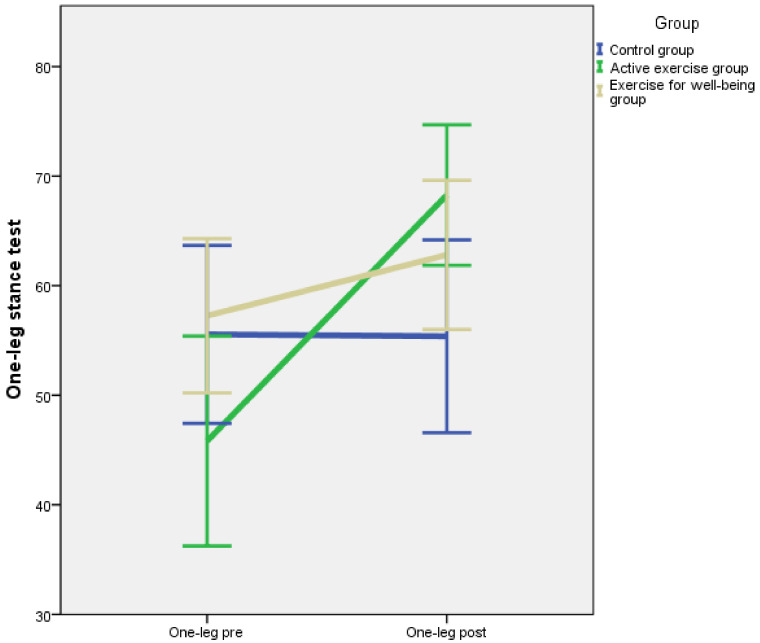
Changes in the one-leg stance test.

Baseline and post-intervention outcome measurements divided by intervention groups are summarised in Table 2. According to the results of the one-way ANOVA, there were no significant differences between groups for flexibility, centre of gravity, S-FIQ, VAS and one-leg stance test (*p* = 0.379, *p* = 0.669, *p* = 0.667, *p* = 0.237, *p* = 0.103, respectively). A repeated measures model was applied, and the *p*-value corresponding to the interaction between the inter-group factor and the intra-group factor is shown in Table 2. The pre-post intervention evolution for each outcome is illustrated in Figure 2, Figure 3, Figure 4, Figure 5 and Figure 6.

We observed a tendency to experience a slight or strong improvement in many outcomes, even for the control group. In other words, the patients seemed to perform spontaneously better at the second measurement. This could be explained by the training effect of the interventions. However, after applying a repeated measures model, no significant interactions between group and evolution for flexibility (*p* = 0.193) nor for centre of gravity (*p* = 0.184) were found. Moreover, the difference between groups was not significant (*p* = 0.632, *p* = 0.745, respectively).

However, we found a significant interaction for the S-FIQ (*p* = 0.002, η^2^ = 0.129), the VAS (*p* = 0.020, η^2^ = 0.084) and the one-leg stance test (*p* = 0.002, η^2^ = 0.132). These results can be observed in Figure 4, Figure 5 and Figure 6. In a deeper analysis performed with the Tukey HSD post hoc comparison, we could observe an evident improvement of the S-FIQ from baseline for the active exercise group (*p* < 0.001) and the exercise for well-being group (*p* = 0.004). Differences in post treatment measurements between these groups and the control group were close to significant (*p* = 0.057, *p* = 0.061, respectively). In the same way, the post hoc comparison showed significant improvement in the VAS for the active exercise group (*p* = 0.002) and the exercise for well-being group (*p* = 0.006). However, the differences in post intervention measurement in relation to the control group were not significant (*p* = 0.911, *p* = 0.245) according to the post-hoc comparison. Finally, according to the results of the Tukey method, there was a strong improvement in the one-leg stance test for the active exercise programme group (*p* < 0.001) and also a significant improvement for the exercise for well-being group (*p* = 0.025). The difference in the post-intervention measure in relation to the active exercise programme group were no significant (*p* = 0.123, *p* = 0.702).

When comparing the active exercise programme with the exercise for well-being, we observed (Table 2) a better performance of the first group, at least in descriptive terms. Nevertheless, we did not find any significant differences between both groups. It would be interesting to assess if the other observed differences would become significant with bigger samples.

Lastly, in the subjective assessment of fatigue experienced during the sessions, the mean for the active exercise programme group was 6.30 ± 1.88 and 5.52 ± 1.55 for the exercise for well-being group. These differences were not significant.

## 4. Discussion

The results of this study indicate that the active exercise programme and the exercise for well-being improve static balance, flexibility and pain in women with fibromyalgia compared to the control group. The most significant improvements were found in the active exercise programme group. In order to facilitate the discussion, this section is structured by the outcome measures analysed in the study.

### 4.1. Balance

The exercise for well-being group showed improvements in the final static balance scores (one leg stance test). However, these changes were not significant. In contrast, the active exercise programme group obtained significant improvements in static balance. We suspect that this improvement was due to the type of balance exercise performed. The women in the active exercise programme group did static balance exercises, maintaining equilibrium for 10 s. The active exercise programme group repeated the exercise slowly with eyes open and closed while the exercise for well-being exercises were performed with eyes open only.

With respect to the posturograph used (Wii-Fit, Nintendo ©), the results of the medical evidence have supported its use. In relation to its validity, several investigations can be highlighted. Holmes et al. [41] and Meldrum et al. [42] assessed balance in patients with neurological disease using the Wii-Fit (Nintendo ©). They concluded that the platform is a valid tool for the quantification of the postural stability, it is easy to use [41] and has no adverse effects when it is used to assess balance impairment [42]. Additionally, in agreement with Huurnink et al. [43], their results showed that it is a sufficiently accurate platform to quantify the centre of pressure trajectories in single-leg balance exercises.

Although there are few studies on active exercise programmes and balance in patients with fibromyalgia, the available literature has shown how physical therapy can improve balance in patients with this condition. The research conducted by Espí et al. [44] in 2016 analysed the effect of therapeutic aerobic exercise in women with fibromyalgia and concluded that exercise improves general discomfort. In addition, the authors observed that the effectiveness was greater when the exercise was combined with music therapy, which led to further improvements in quality of life and balance. Moreover, Kibar et al. [45] carried out an active exercise programme based on flexibility exercises to improve balance in these patients and observed a beneficial effect on static balance and functional levels. A study published in 2020 [46] analysed the effects of a 5-week core stability active exercise programme. The results showed that this exercise modality improved dynamic balance and postural control in women with fibromyalgia.

Based on the results of the present study and with those obtained by the mentioned authors, we consider that the active exercise interventions can improve balance in women with fibromyalgia.

As for exercise for well-being therapy, Roger et al. [25] specified that few studies applied this treatment approach and assessed its effects on balance of women with fibromyalgia. The authors observed that this treatment approach generally improved balance, but its potential to decrease falls had to be clarified.

However, there is evidence of the use of other exercise modalities for the improvement of balance, such as yoga or tai chi. Ulger et al. [47], in 2011, showed that yoga has a positive effect on women with balance and gait disorders due to musculoskeletal problems. In addition, Wong et al. [48] carried out a study in 2018, and showed how a 12-week tai chi intervention was effective in improving balance, fatigue, strength and flexibility in women with fibromyalgia.

As for the variable centre of gravity, we obtained non-significant results using the one leg stance test (Wii-Fit, Nintendo ©) after applying the experimental treatments. In the literature, we have not found any research assessing the changes of the centre of gravity in women with fibromyalgia nor studies that compared active exercise programmes or exercises for well-being. Only one study that analysed this variable and compared an active exercise programme with acupuncture and a control group was found [46]. The results coincide with ours, as the centre of gravity did not experience statistically significant improvements after both treatments. The authors concluded that neither the centre of gravity position nor the one-leg stance test were influenced by the intervention received in any of the groups.

### 4.2. Flexibility

The results of the Sit and Reach test showed a significant improvement in the flexibility of the participants of both experimental groups, the active exercise programme and the exercise for well-being groups. However, the improvements were more marked in the active exercise programme group. Our findings coincide with other research, such as that of Valencia et al. [49], who analysed the short- and medium-term effect of an active exercise programme on pain perception and muscle flexibility. Valencia et al. [49] showed how 20 women with fibromyalgia improved their level of flexibility and general well-being after an intervention based on kinesio-therapy and stretching exercises. The treatments were applied twice a week for 12 weeks and the outcome measurement were completed pre and post intervention and at 24 weeks of follow-up after the end of the treatments.

In the study conducted by Jones et al. [50], the objective was to assess the efficacy of a muscle strengthening programme compared to a stretching programme. A total of 68 women with fibromyalgia completed two weekly treatment sessions over 12 weeks. The authors found that flexibility improved with the stretching programme.

In addition, Ayan et al. [51] evaluated the long- and short-term effects of a multimodal programme (one hour every week for 3 months) combining muscular endurance and flexibility exercises with breathing and relaxation techniques plus a half-hour active exercise session. The sample consisted of 21 women with fibromyalgia that were assessed at baseline, post treatment and at 6 months of follow-up after the end of treatment. The authors demonstrated how flexibility exercises with breathing and relaxation techniques, in addition to active exercises, improved flexibility and reduced the impact of the disease. We consider it important to highlight that the duration of the treatment was longer in the studies conducted by Valencia et al. [49], Jones et al. [50] and Ayan et al. [51] than in our study, although the results related to flexibility coincide.

### 4.3. Perceived Exertion

No statistically significant changes were found in relation to the perceived exertion measured with the Borg scale or the subjective feeling of tiredness during the sessions. We were not able to conclude which experimental group (exercise for well-being or active exercise programme) had a significant lower level of perceived exertion. However, we found that the mean score for the exercise for well-being group was lower, 5.52 ± 1.55, (and therefore, better), than for the active exercise programme group.

The lower score obtained in the exercise for well-being group could be due to the fact that this therapy is carried out by the participants slowly, in a relaxed manner and with greater concentration. According to the bibliography consulted, there is no conclusive data in relation to the Borg scale in studies that carry out active exercise programmes and exercise for well-being treatments. There is scarce scientific evidence with a methodology similar to the one developed in our study. The study conducted by Nielens et al. [52] is one of them. The authors assessed the cardiorespiratory capacity and the perceived effort when performing a fitness programme, comparing 30 women with fibromyalgia syndrome and 67 healthy women. Nielens et al. [52] concluded that perceived exertion is greater in patients with fibromyalgia than in healthy patients. These results confirm that women with fibromyalgia have a higher perceived exertion than healthy patients.

### 4.4. Pain and Quality of Life

We believe that all the improvements in terms of flexibility and balance must have influenced the pain and improvement in the quality of life perceived by the patients, as the final pain scores obtained in both the active exercise programme group and the exercise for well-being group were lower. In this respect, we coincide with the studies carried out by Castro-Sánchez et al. [53], Kesiktas et al. [54] and Matsutani et al. [55]. All of them showed how stretching is effective for pain relief in patients with fibromyalgia. Moreover, Busch et al. [17] concluded that short-term aerobic exercise in fibromyalgia patients improves pain, global sense of well-being and physical function. Other studies confirmed that low-intensity, individualised physical exercise improves function and reduces symptoms of fibromyalgia [18]. In addition, Hooten et al. [56] demonstrated that, in two groups of 36 fibromyalgia patients, 3 weeks of aerobic exercise and strengthening exercise had similar effects on pain relief.

A study that was carried out by Yang et al. a [57] showed how a 4-week exercise for well-being programme (a treatment period that coincides with our study) helped to improve chronic pain and mood disorders.

Chen et al. [58] showed how exercise for well-being treatment can be very effective in treating pain and associated symptoms in fibromyalgia patients. Ten women who completed 5 to 7 exercise for well-being sessions of 40 min duration for more than 3 weeks were evaluated at baseline, post treatment and at 3 months of follow-up after the end of treatment. However, the methodological quality is questionable as the sample size was very small and had no control group.

In any future research, we would recommend that active exercise and exercise for well-being were combined to assess whether better improvements could be achieved. We would suggest increasing the duration of the treatment and including a follow-up period. It would also be interesting to carry out studies in which different types of exercise for well-being, such as tai chi or yoga, are practised. This would allow us to ascertain their effects on the variables studied in this population. On the other hand, it would be advisable in future studies to take into account variables such as body mass index and to monitor the presence of menopause. Research has shown that age [59] and body mass index [60] can influence musculoskeletal symptoms. In this regard, the prevalence of musculoskeletal disorders increases with age and appears to be associated with menopause [61]. On the other hand, a higher body mass index may be associated with greater pain and disease severity in patients with musculoskeletal disorders associated to fibromyalgia. It would also be interesting to provide a correlation with the pressure pain thresholds according to the American College of Rheumatology criteria.

### 4.5. Limitations of the Study

One limitation was the difficulty of learning each of the exercises to be performed. In the case of the exercise for well-being group, as this exercise is still quite unknown in our environment, it was necessary to explain the three essential aspects of its practice. These include the control of the position of the body and breathing as well as the regulation of the mind. Additionally, each exercise needed to be repeated during the learning process, and sometimes, it was necessary to rest during the practice of the exercises. Therefore, we consider that the four weeks of duration of the experimental treatment may be insufficient to obtain all the expected benefits, and perhaps a previous learning period would have been necessary.

However, if the treatment period was lengthened, the non-compliance with the programme could increase. Our research group has conducted a previous a study, with the same fibromyalgia associations, which studied the effects of the moderate consumption of red wine in these patients [62]. Out of 80 participants, there were 33 losses due to non-compliance (20 in the control group and 23 in the experimental group) in a 4-week intervention. Likewise, in the literature, we found studies carried out with this population [63,64] which had patient participation and follow-up dropout rates similar to the data provided in our research. The losses are generally due to the fluctuation of symptoms and the effects of different factors that can affect the condition [65]. Therefore, the longer the treatment period is, the higher the possibility that a participant stops the treatment or does not attend the measurement sessions. However, in this study, we have a final sample number of 93 women diagnosed with fibromyalgia, which is higher than the studies cited above.

## 5. Conclusions

The results of the present study indicate that active exercise and exercise for well-being improve pain, flexibility, static balance and quality of life in women with fibromyalgia. However, the active exercise programme achieved better results than the exercise for well-being. No statistically significant differences were found between groups in relation to the perceived feeling of tiredness during the sessions.

## Figures and Tables

**Table 1:** Socio-demographic characteristics of the sample.

Outcomes		*N*
**Working status**	Housewife	41
	Unemployed	10
	Employed	23
	Incapacitated	18
	Retired	1
**Marital status**	Married	81
	Lives with her partner	2
	Single	2
	Separated	2
	Divorced	3
	Widow	3
**Education level**	With no studies	11
	Primary Education	40
	Secondary Education	23
	Bachelor’s Degree	19
**Smoking habits**	No	25
	Yes	68

**Table 2:** Baseline and results of the post-intervention outcome measures.

Baseline Outcomes		Mean ±SD			*p*-Value *
		CG (*N* = 29)	AEG (*N* = 33)	EWG (*N* = 31)	
Flexibility	Pre	−6.24 ± 11.01	−9.24 ± 9.37	−6.10 ± 9.96	0.193
	Post	−3.14 ± 9.08	−2.94 ±10.51	−2.03 ± 10.80	
Centre of gravity	Pre	54.07 ± 3.58	53.39 ± 2.99	53.94 ± 2.93	0.184
	Post	53.10 ± 2.64	54.30 ± 5.78	52.74 ± 2.66	
S-FIQ	Pre	68.86 ± 13.34	67.21 ± 16.51	65.35 ± 14.95	0.002
	Post	69.45 ± 4.02	57.79 ± 17.95	57.71 ± 15.79	
VAS	Pre	7.34 ± 1.61	7.88 ± 1.58	7.16 ± 2.02	0.020
	Post	7.31 ±1.93	6.79 ± 1.43	6.16 ± 2.56	
One-leg stance test	Pre	55.55 ± 21.35	45.82 ± 27.02	57.26 ± 19.20	0.002
	Post	55.38 ± 23.14	68.27 ± 18.08	62.81 ± 18.56	

Note: CG: Control group; AEG: Active exercise Group; EWG: Exercise for well-being group; S-FIQ: Spanish Fibromyalgia Impact Questionnaire; VAS: Visual analogue scale, pre: before intervention, post: after intervention. * *p*-value corresponding to interaction contrast, according to a repeated measures model. A significant result means that change pre–post depends on the treatment.

## Data Availability

The data underlying this article cannot be shared publicly to maintain the privacy of individuals that participated in the study. The data will be shared on reasonable request to the corresponding author.

## Supplementary Materials

The following are available online at https://www.mdpi.com/article/10.3390/jcm10173826/s1, Figure S1: Exercise 14: The carpenter handles a drill, Figure S2: Exercise 17: The white crane circles its knees, Figure S3: Active exercise programme.
