# Supplementary material for: Effects of Non-Pharmacological Treatment on Pain, Flexibility, Balance and Quality of Life in Women with Fibromyalgia: A Randomised Clinical Trial
Source: J Clin Med. 2021 Aug 26;10(17):3826. doi: 10.3390/jcm10173826 (PMC8432021; doi:10.3390/jcm10173826)
Supplement: Supplementary file 1 [file jcm-10-03826-s001.zip › jcm-1342374-proofed-supplementary.pdf]

SUPLEMENTAL MATERIAL

1. Exercises for well-being programme

The exercises performed during the sessions were the ‘twenty Wang Ziping figures for health and longevity’ of Qi Gong described by ‘Wang Ziping’.

According to the Chinesse Traditional Medicine, the names of the exercises were: exercise 1: Breath out the bad air and breath in fresh air; exercise 2: the children receive food; exercise 3: the phoenix tightens its grip; exercise 4: Tension the arc in two directions; exercise 5: The king raises the tripod; exercise 6: Raise your arms to pull the fruit out; exercise 7: Look for the bottom of the sea; exercise 8:The rhinoceros looks at the moon; exercise 9: Lotus leaves swing in the wind; exercise 10: Push a stone tablet; exercise 11: Introduce a hand in Mount Huashan; exercise 12: Put aside the mane of a white horse; exercise 13: The phoenix spreads its wings; exercise 14: The carpenter handles a drill; exercise 15: The black dragon turns around; exercise 16: The Arat dominates a tiger; exercise 17: The white crane circles its knees; exercise 18: The friar squats; exercise 19: Kick with the lower half of the legs; exercise 20: The fairy walks back and forth.

Reference:

Ziping W. Wushu. 20 exercises for longevity (20 ejercicios para la longevidad). Miraguano. Madrid (Spain) Miraguano;1988.

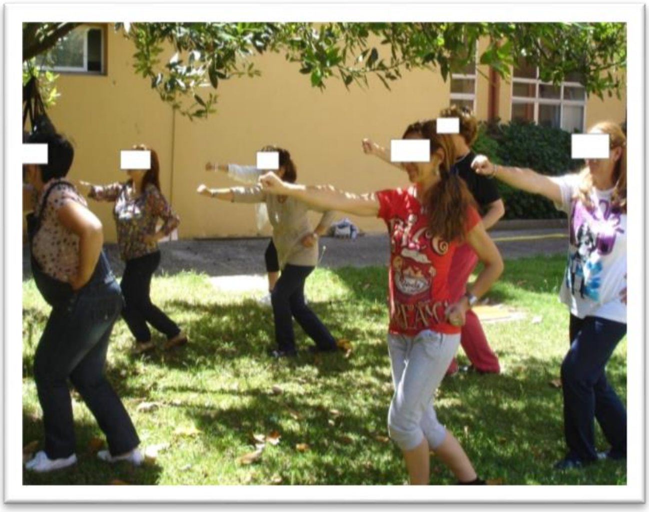

Figure S1. Exercise 14: The carpenter handles a drill

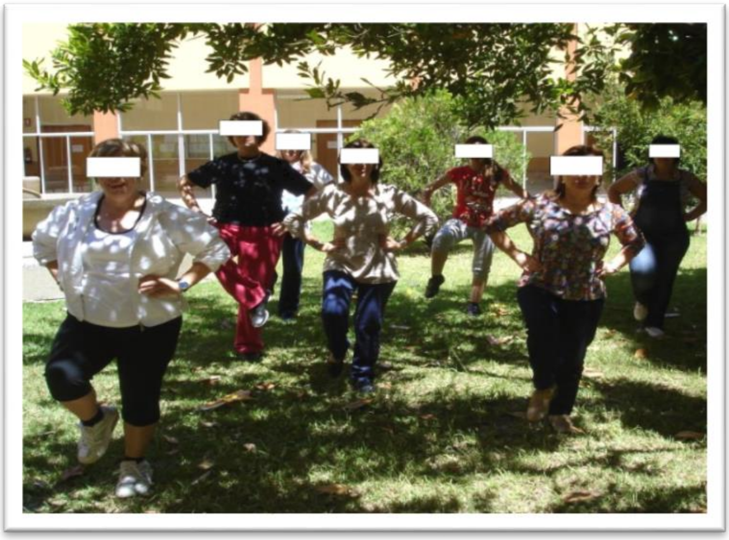

Figure S2. Exercise 17: The white crane circles its knees

2. **Active exercise program.**

The exercise programme included a warm up of 3 to 5 minutes walking, active mobilisation exercises of the shoulders, spine and hips, static balance exercises and stretches. The shoulder, hip and cervical spine exercises were performed in standing. The thoracic spine and lumbar spine were done on an exercise mat and in sitting. All exercises were performed in coordination with controlled gentle breathing. Each mobilisation exercise was done at maximum range of movement, was maintained for 10 seconds and repeated 6 times with eyes open and closed. All movements were done slowly and avoiding pain and fatigue. The exercises performed were the following:

Active shoulder exercises:
- Unilateral shoulder flexion.
- Bilateral shoulder flexion.
- Self-assisted shoulder flexion.
- Unilateral shoulder extension.
- Unilateral shoulder abduction.
- Bilateral shoulder abduction.
- Unilateral shoulder adduction.
- Unilateral shoulder internal rotation.
- Unilateral shoulder external rotation.
- Unilateral shoulder tennis stroke.
- Unilateral shoulder tennis stroke (backhand).

Active HIP exercises:
- Hip flexion.
- Hip extension.
- Hip abduction.
- Hip adduction.
- Flexion associated with hip abduction.
- Flexion associated with hip adduction.
- Internal rotation of the hip.
- External rotation of the hip.

Active exercises of the cervical spine:
- Cervical spine flexion. Flexion movements, until the chin touches the chest.
- Cervical spine extension. Extension movements, bringing the head backwards.
- Cervical spine lateral: Movement of the head to both sides, trying to touch the shoulder with the corresponding ear.
- Cervical spine rotation: Turning of the head to both sides, trying to bring the chin in contact with the corresponding shoulder.

- Turn the head in a clockwise direction. Repeat the exercise in the opposite direction. The movements should be done slowly to avoid possible dizziness.

Active exercises of the thoracic spine:

- Patient lying supine with hands on the back of the neck and fingers interlocked. Elbows in contact with the floor. Raise the elbows to bring them as close together as possible, without unlacing the fingers of the hands, and at the same time exhale deeply, expelling air through the mouth. Return to the initial position and repeat the exercise.
- Patient in the supine position with arms extended along the body. Flex the legs and thighs until they come into contact with the abdominal wall. Return to the initial position.
- Patient in dorsal decubitus position, with knees bent. Feet flat on the floor and arms crossed over the chest. Maximum lifting of the buttocks, slowly, without arousing pain. Return to the initial position.
- Patient in standing position, with feet parallel. Maximum knee flexion, leaning the trunk forwards, until the tips of the toes touch the floor. Return to the initial position.

Active exercises of the thoracic and lumbar spine:

- Patient in sitting. Legs together and extended, touch with both hands in the direction of the feet, as far as possible.
- Patient in supine position. Starting position with the legs extended. Bend one leg and embrace it without touching the floor (alternately).
- Patient in supine position. Starting position with the legs extended. Bend both legs and embrace them without touching the floor.
- Patient in supine position. Trunk rotation with shoulders in abduction on the floor, touch the knee with the opposite hand. Alternate right and left.
- Patient in sitting with the hips in abduction, touch with both hands the middle area of the floor between the two legs.
- Patient in sitting. Crossed legs, arms raised, touch hands to the floor.
- Patient in sitting. With legs open, lower arms crossed, and touch with the hand to the opposite foot.
- Patient in supine position. Knees bent, hands behind head, touch knees with hands.
- Patient in supine position. Lower and raise the legs with the hip and the knee in flexion. Alternate right and left lower limb.

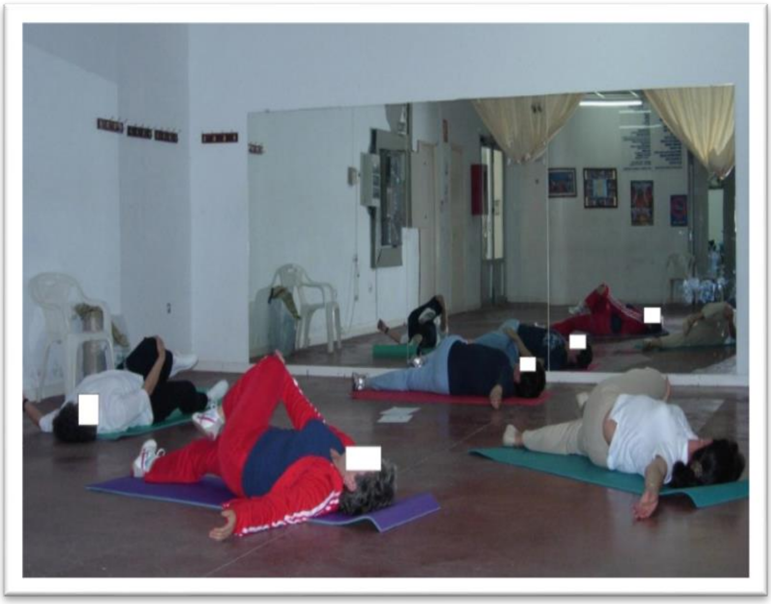

Figure S3. Active exercise programme
